# A rare case of bile leak due to type 2 duct of Luschka injury post open cholecystectomy: a case report

**DOI:** 10.1093/jscr/rjae179

**Published:** 2024-03-21

**Authors:** Jiksa Muleta, Eden Belayneh, Kalkidan Haile, Amanuel Worku

**Affiliations:** Department of Internal Medicine, Myungsung Medical College/MCM Comprehensive Specialized Hospital, Addis Ababa, Ethiopia; Department of Internal Medicine, Myungsung Medical College/MCM Comprehensive Specialized Hospital, Addis Ababa, Ethiopia; Department of Internal Medicine, Myungsung Medical College/MCM Comprehensive Specialized Hospital, Addis Ababa, Ethiopia; Department of Internal Medicine, Myungsung Medical College/MCM Comprehensive Specialized Hospital, Addis Ababa, Ethiopia

**Keywords:** bile leak, biloma, cholecystectomy, duct of luschka, ERCP, case report

## Abstract

Bile leak is an uncommon complication post cholecystectomy. The bile may originate from the cystic duct stump and less commonly from the aberrant ducts of Luschka. Such complications may occur when anatomical variations in the biliary tree go unnoticed. This case report presents a 24-year-old otherwise healthy female who presented with abdominal pain and distension that began 3 days after she underwent open cholecystectomy for symptomatic cholelithiasis. Imaging revealed choledocholelithiasis in the distal common bile duct, and free intrabdominal fluid collection. Endoscopic retrograde cholangiopancreatography done showed contrast leak from the duct of Luschka to the gall bladder bed. The biliary tree has many anatomic variations. These variations have clinical significance for surgical treatment of patients with biliary pathology. Surgeons should be aware of such variations to decrease the risk of bile leak post cholecystectomy.

## Introduction

The incidence of bile leak after laparoscopic cholecystectomy appears to be higher than for open cholecystectomy. It has been estimated at 1.1% to 4.0%. The majority of bile leaks following cholecystectomy are from two sources: the cystic duct stump and aberrant branches of hepatic ducts, including ducts of Luschka [1]. Serious complications post cholecystectomy includes bile duct injury (BDI) including bile leaks and peritonitis [2].

There are several classifications of BDI. The most known classification system is the ‘Strasberg classification system’, which classifies BDIs into the following five categories. Type A: Bile leak from the cystic duct or liver bed; Type B: Partial occlusion of the biliary tree, commonly of an aberrant right hepatic duct (RHD); Type C: Bile leak from aberrant RHD that is not communicating with the common bile duct (CBD); Type D: Lateral injury of the biliary system; and Type E: Circumferential injury of the biliary tree with loss of continuity. From these categories, injury to Type A leads to bile leakage from the cystic duct remnant or the bile ducts of lushcka [3].

The major presenting symptoms of bile leak are jaundice, abdominal pain, nausea, pyrexia, and abdominal distension. Investigations include routine hematological and biochemistry tests, including liver function tests followed by imaging with abdominal ultrasound (US), computed tomography (CT) scan, or magnetic resonance imaging (MRI)/magnetic resonance cholangiopancreatography (MRCP) [4]. Management options include insertion of biliary stent across the ampulla at the time of endoscopic retrograde cholangiopancreatography (ERCP) to decrease pressure in the proximal biliary system or performing sphincterotomy which promotes free flow of bile across the ampulla without stent insertion [5].

## Case report

A 24-year-old otherwise healthy female presented with abdominal pain and distension of 5 days duration. The symptoms started 3 days after she underwent open cholecystectomy for symptomatic cholelithiasis. The open cholecystectomy was done at the referring hospital where laparascopic cholecystectomy was not practiced. Preoperative abdominal US showed cholelithiasis with no CBD dilation or choledocholelithiasis. She had associated fever, chills, nausea, and vomiting. Additionally, she complained of yellowish discoloration of the eyes, loss of appetite, and fatigue. She was given antibiotics at the referring hospital but did not show improvement.

On physical examination patient appeared sick looking with the following vital signs: B.P-100/60 mmHg, P.R-112 bpm, R.R-22 bpm, Temperature-36, and Spo2-95% on room air. She had an icteric sclera and tenderness over the right upper quadrant area of the abdomen. Laboratory Investigation revealed CBC of 10 360 with neutrophil count of 78.8%, LFT showed Total Bilirubin of 2, ALP was three times elevated, GGT was four times elevated, RFT was normal, serum potassium was 3.2, CRP was >200, and INR was 1.3.

Subsequently, MRCP and abdominal CT scan with contrast was done and showed choledocholelithiasis in the distal CBD, and free intrabdominal fluid collection possibly because of bile leak from cystic duct stump. Patient was then referred to our hospital for better management after 10 days of symptom onset.

**Figure 1:**
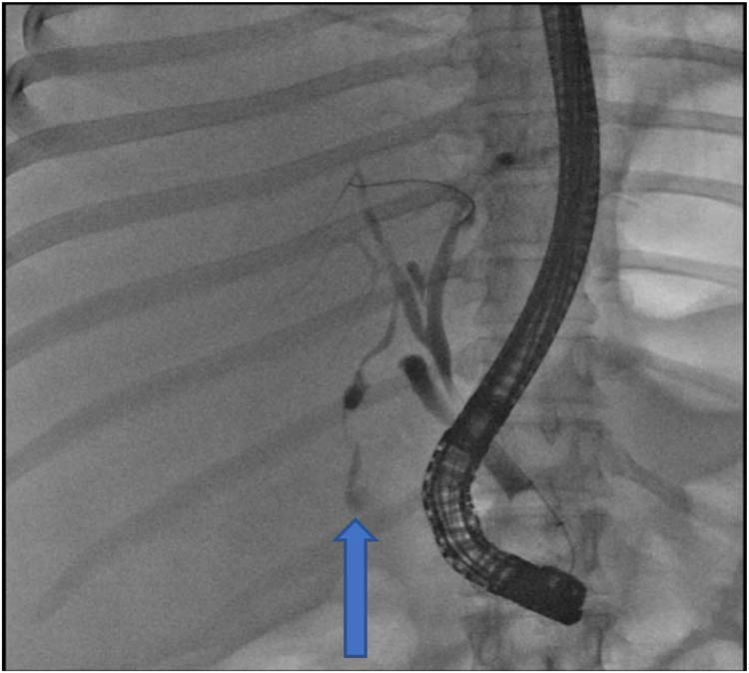
Fluoroscopy image indicating bile leak from type 2 duct of Luschka.

**Figure 2:**
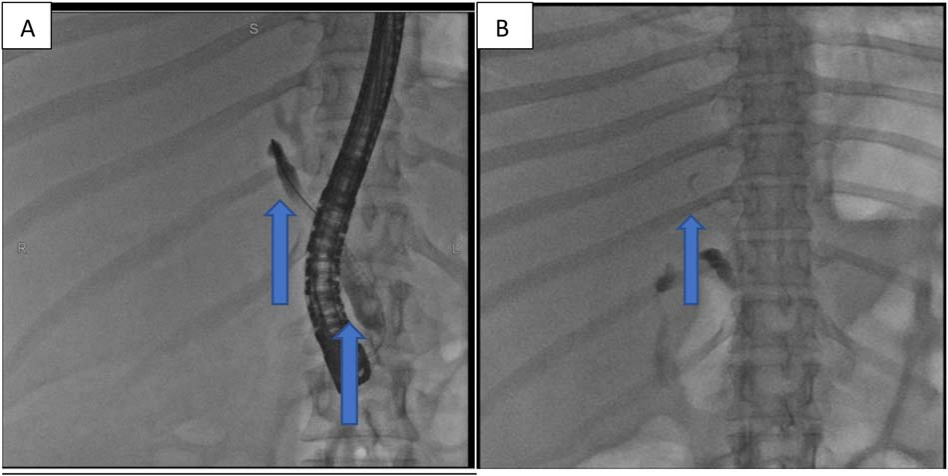
(A) First fluoroscopic image shows evidence of no bile leak from cystic duct stump as indicated by the arrows; (B) second fluoroscopic image shows a stent placed into the RHD.

At our facility, ERCP was done. The bile duct was cannulated with 0.035 wire-guided sphincterotome. Cholangiogram showed normal caliber biliary tree with one filling defect in the distal CBD and contrast leak from duct of Luschka to the gall bladder bed (Fig. 1). There was no contrast leak from the cystic duct stump (Fig. 2A). Standard sphincterotomy was performed and a single mulberry stone was removed using stone retreival balloon and double pig tail biliary plastic stent was placed into the RHD and good bile flow was achieved post procedure (Fig. 2B).

**Figure 3:**
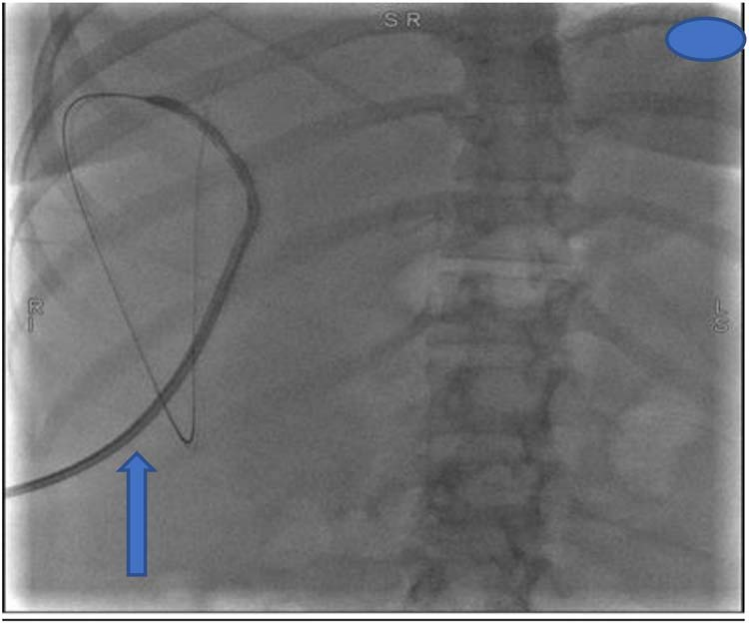
Insertion of percutaneous catheter drainage for drainage of biloma.

The following day, percutaneous drainage of the biloma was done through a pigtail catheter inserted through sub phrenic space access (Fig. 3). Subsequently after 6 days, drainage amount of the biloma markedly decreased and catheter was removed on the seventh day after insertion. The patient had marked symptom improvement afterwards, and control abdominal US was normal.

## Discussion

The normal anatomy of the biliary tree is observed in only 58% of the population [6]. This indicates biliary anatomic variations to be a common occurrence, holding clinical significance. Aberrant biliary ducts in the gallbladder bed, known as Ducts of Luschka, were originally documented by the German anatomist Hubert von Luschka. Two different types of these ducts have been identified: the first type draining directly into the gallbladder (type 1), and the second one running along the gallbladder bed and draining into the hepatic duct (type 2) [7].

Clinical presentation of patients with postoperative bile leakages varies from asymptomatic to biloma formation and biliary peritonitis with sepsis. However, in most cases, patients will express symptoms that are more severe than anticipated for the postoperative course, typically occurring within the initial week following the operation [8, 9]. In our case, patient developed abdominal distension, pain and fever 3 days after she underwent open cholecystectomy. Initially symptoms were attributed to normal post op course but as she started to have yellowish discoloration of eyes and symptom worsening further investigations were carried out.

Because of their clinical significance, imaging of ducts of Luschka becomes important and can be done in the preoperative, intraoperative, and postoperative period [10]. Due to the availability of multi-detector CT and MRI, particularly with the advent of MRCP, the diagnosis of leaking ducts of Luschka is made both feasible and highly accurate without the need for interventional techniques like ERCP for diagnostic purposes only [2]. In this case, initial imaging with CT and MRCP revealed bile leakage, fluid collection in the abdomen and choledocholelithiasis. These findings prompted the use of ERCP for intervention.

ERCP is an effective and safe modality for diagnosing and treating bile leaks following cholecystectomy [11]. Sphincterotomy lowers the transpapillary pressure gradient by opening the ampullary orifice. Additionally, stenting if feasible facilitates bridging of the leak site, promoting healing of the injured area [12]. Similarly, in our case the contrast leak from duct of Luschka to the gall bladder bed was detected during ERCP. Subsequently, standard sphincterotomy was done and plastic stent was placed into RHD to achieve bile flow. The following day a catheter was placed to drain the remaining biloma by the interventional radiologists. Our patient had marked symptom improvement post the procedure.

A similar case report in 2019 presented a 78-year-old male who developed bile leak at fourth postop day after undergoing open cholecystectomy for cholelithiasis. ERCP showed no contrast leak from the cystic duct however leakage from the ducts of lushcka were witnessed. Following sphincterotomy, a biliary stent and drainage tube were placed. Patient was discharged free of symptoms on post-op day 12 [10]. The above mentioned case is similar to ours in many aspects. Both patients had open cholecystectomy and later developed bile leak. The site of biliary leakage was confirmed by ERCP in both cases and their management included sphincterotomy and biliary stenting.

Another case report published in 2022 presented a female who had intense right upper quadrant abdominal pain 7 days post laparascopic cholecystectomy. She was investigated with abdominal and pelvic CT scan, which showed intra-abdominal fluid accumulation. Subsequently, patient underwent exploratory laparoscopy which revealed biliary leakage from the ducts of Luschka. The duct was then sutured and a drain was left in place [13]. The complication presented in this case report is similar to the one our patient had. However, the management was different. While the case above used laparascopy and suturing techniques, our patient underwent ERCP and stent insertion.

## Conclusion

Bile leakage is a rare complication following cholecystectomy. Preoperative imaging assessment of subvesical bile ducts and overall biliary anatomy can help prevent such complications. Surgeons benefit from awareness of potential injuries to these ducts, enabling careful evaluation and intervention during procedures. In case of injuries, identifying the leakage site is crucial, with ERCP commonly employed as a standard management approach.

## Data Availability

All data generated or analyzed during this case report are included in this article.
